# Nonequilibrium phases of a biomolecular condensate facilitated by enzyme activity

**DOI:** 10.1039/d5sm01106j

**Published:** 2026-03-12

**Authors:** Sebastian Coupe, Nikta Fakhri

**Affiliations:** a Department of Biology, Massachusetts Institute of Technology Cambridge MA USA; b Department of Physics, Massachusetts Institute of Technology Cambridge MA USA fakhri@mit.edu; c Bioengineering Department, Stanford University Stanford CA USA

## Abstract

Biomolecular condensates represent a frontier in cellular organization, existing as dynamic macromolecular structures driven out of equilibrium by active cellular processes. Here we explore active mechanisms of condensate regulation by examining the interplay between DEAD-box helicase activity and RNA base-pairing interactions within a reconstituted ribonucleoprotein condensate. We demonstrate that the ATP-dependent activity of a DEAD-box helicase—a key class of enzymes in condensate regulation—acts as a nonequilibrium driver of condensate properties through the continuous remodeling of RNA interactions. By combining the LAF-1 DEAD-box helicase with a designer RNA hairpin concatemer, we unveil a complex landscape of dynamic behaviors, including time-dependent alterations in RNA partitioning, evolving condensate morphologies, and shifting condensate dynamics. Importantly, we reveal an antagonistic relationship between RNA secondary structure and helicase activity which enables an initially homogeneous nonequilibrium state. By elucidating these nonequilibrium mechanisms, we gain a deeper understanding of cellular organization and expand the potential for active synthetic condensate systems.

## Abstract 2

Energy-dependent helicase activity and RNA secondary structure work together to preserve an initial nonequilibrium state of condensate homogeneity and dynamics.

## Graphical abstract

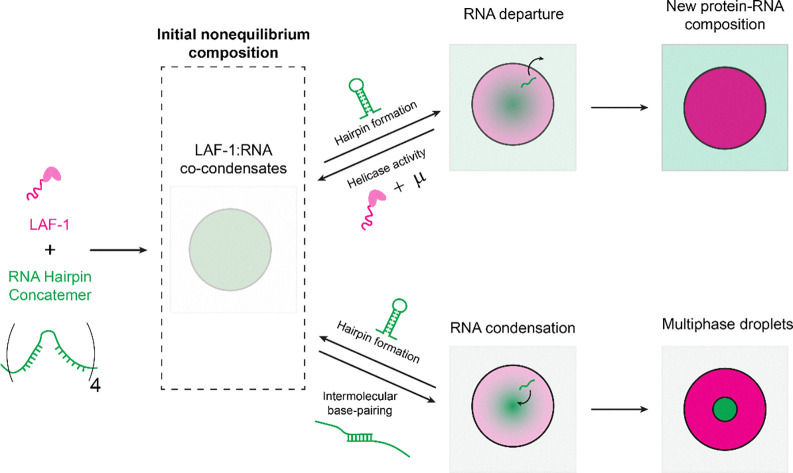

## Introduction

Biomolecular condensates are dynamic intracellular structures that play a crucial role in organizing cellular biochemistry by locally concentrating proteins and ribonucleic acids (RNA).^1–3^ Condensates participate in a range of cellular functions, from accelerating biochemical reactions to sequestering biomolecules and regulating biopolymer concentrations in the dilute phase.^1–3^ While the principles of equilibrium polymer phase separation have been instrumental in describing many aspects of condensate formation and phase behavior, it is essential to recognize that these structures operate within a nonequilibrium cellular environment. As a result, biomolecular condensates are subject to kinetic trapping, aging effects,^4–7^ and can be driven out of equilibrium by chemically active processes such as post-translational modifications, polymer production and degradation, and active remodeling of intermolecular interactions.^8–14^ Active nonequilibrium driving leads to novel behaviors in condensates, including size uniformity and resistance to Ostwald ripening,^15–17^ asymmetric localization patterns,^18^ complex topologies,^19,20^ dissolution,^12,13^ and even droplet division and motion.^16,21,22^ A deeper understanding of the interplay between active biochemical processes and the properties of biomolecular condensates is critical for manipulating these structures within cells and designing synthetic active condensates. Additionally, these active processes are fundamental to defining the evolutionary constraints and strategies that govern biomolecular condensation in cells.

DEAD-box helicases are a class of enzymes whose activity is central to biomolecular condensate regulation and homeostasis.^12,23^ The canonical biochemical role of DEAD-box helicases is in RNA processing, where they remodel RNA secondary structures and intermolecular interactions.^24,25^ DEAD-box helicases are non-processive RNA helicases that locally destabilize RNA base-pairing interactions, using adenosine triphosphate (ATP) hydrolysis to facilitate their cycling on RNA.^24,25^ More recently, these enzymes have gained interest due to their association with biomolecular condensates and the role their activity plays in facilitating normal condensate localization and structure in cells.^12,13,23,26,27^ Their activity has also been tied to condensate dissolution and dynamics.^12,13,23,28–30^ The mechanistic roles these proteins play in dictating condensate properties is an open area of interest and the capability of DEAD-box helicase activity to produce novel condensate behaviors is only starting to be realized.

Many biomolecular condensates are enriched in RNA and rely on RNA for their formation.^31^ While RNA can fluidize biomolecular condensates in some contexts,^32–34^ RNA base-pairing interactions can also drive condensate formation,^35^ with extensive base-pairing shown to decrease condensate dynamics.^36,37^ Additionally, RNA secondary structures can influence RNA incorporation into biomolecular condensates, distribution of RNA within the condensed phase, and the dynamics of the condensates themselves.^35,38–41^ This suggests that condensates containing RNA base-pairing interactions can be sustained out of equilibrium when coupled with energy-dependent helicase activity, which modifies RNA secondary structures and alters RNA–RNA interaction strengths.^27^

To investigate the interplay between RNA–RNA interactions, DEAD-box helicase activity, and condensate properties, we engineered a reconstituted system composed of the LAF-1 DEAD-box helicase and a simple, repetitive, base-pairing RNA—a modified MS2 hairpin concatemer. Our results demonstrate that this system exhibits complex RNA concentration dynamics and gradients that are dependent on LAF-1 helicase activity. Increased helicase activity enhances RNA mobility within the condensed phase and prevents the formation of an RNA network. These findings establish a direct link between helicase activity and the metastable composition and dynamical state of condensates. Furthermore, disrupting defined RNA secondary structures induces a second RNA phase transition at the center of the LAF-1 condensed phase. This highlights how ATP-dependent enzymatic activity and RNA secondary structure counterbalance each other to maintain the system in an initially homogenous, nonequilibrium state. Our work broadens the design principles of active synthetic and biological condensate systems and underscores the pivotal role of DEAD-box helicase activity as a nonequilibrium driver of ribonucleoprotein condensate properties.

## Results

### A model condensate system containing a base-pairing RNA and a DEAD-box helicase exhibits time- and activity-dependent RNA gradients

**Fig. 1:**
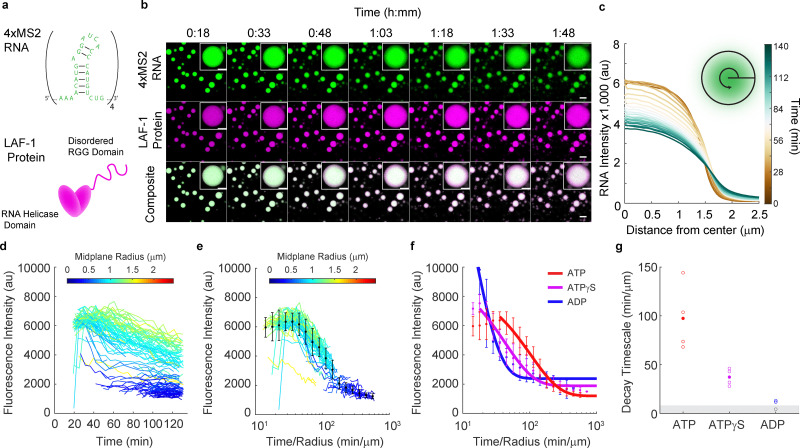
A ribonucleoprotein condensate exhibits time- and activity-dependent RNA gradients. (a) Ribonucleoprotein condensates were formed from a 4xMS2-hairpin concatemer (top) and the LAF-1 DEAD-box RNA helicase (bottom) in the presence of ATP. (b) LAF-1–4xMS2 RNA co-condensates formed in the presence of ATP are initially homogeneous, but over time RNA gradients develop and RNA fluorescence intensity decreases within the condensed phase. (Top) RNA in the condensed phase produces radial gradients over time that depend on condensate size. (Middle) Protein signal within the condensed phase is homogeneous and accumulates in the condensed phase over time. (Bottom) Overlay of the channels shows radial-dependent RNA : LAF-1 ratios, with higher RNA to protein ratios at the center of the co-condensates. Scale bar = 5 µm. The inset depicts these same phenomena for a single condensate, scale bar = 2 µm. (c) Average radial fluorescence intensity profiles for circular droplets with a radius of 1.956 µm ± 0.122 µm. Eleven droplets were included in the average. (d) Average RNA fluorescence at the center of condensates of different sizes was tracked over time, with each trace color-coded by the radius of the condensate's midplane. All condensates experience a decrease in RNA concentration over time, though smaller condensates lose RNA faster, consistent with departure of RNA from the condensate surface. (e) Renormalizing time by each condensate's radius collapses the RNA fluorescence decay curves, consistent with a surface-area-to-volume-ratio dependent process. (f) Renormalized fluorescence curves for LAF-1–4xMS2 RNA condensates formed in the presence of 1.6 mM ATP, ATPγS, and ADP, with an exponential fit to the decay portion of the curve shown in solid lines. As helicase activity is impeded (ATPγS) or eliminated (ADP), RNA departs more rapidly from the condensed phase. (g) Fluorescence decay timescales extracted from exponential fits to the radius-renormalized fluorescence decay curves. A slower departure of RNA from the condensed phase is seen with higher LAF-1 activity. Open circles represent fit timescales obtained for a single time course and filled circles are the average for that condition. Decay timescales below around 10 min µm^−1^ could not be calculated as the fluorescence had decayed to near the plateau value by the onset of the experiment and are represented as open circles within the grey box.

To examine the interplay between DEAD-box helicase activity and RNA–RNA interactions in defining condensate properties, we generated a model hairpin multimer (4xMS2, Table S3), based on a design used previously to study protein–RNA condensates,^28^ and combined it with a DEAD-box helicase known to form condensates *in vitro*, LAF-1 (Fig. 1a).^29,32^ These two components form protein–RNA co-condensates in the presence of ATP which are initially homogeneous (Fig. 1b). However, over time, 4xMS2 RNA gradients appear within the condensed phase, with higher RNA intensity at the center of the droplets (Fig. 1b, c and Video S1). RNA fluorescence intensity decreases in the condensed phase over time suggesting RNA leaves the condensed phase (Fig. 1b, c and Video S1). These RNA gradients emerge faster in smaller droplets, with smaller condensates also exhibiting a faster decay in RNA fluorescence (Fig. S1). LAF-1 protein fluorescence intensity remains homogeneous within the condensed phase and slightly increases over time (Fig. 1b and Fig. S1). We measured the RNA fluorescence decrease at the center of each condensate as a function of time (Fig. 1d). Smaller droplets experience a faster decrease in RNA fluorescence intensity while larger droplets experience a slower decrease in RNA fluorescence intensity. The presence of gradients that are steeper at the boundary and condensate size-dependent fluorescence decay is consistent with RNA leaving from the droplet surface. This suggests that the process depends on the surface area to volume ratio of the condensate and should be renormalizable by a factor of droplet radius. When we renormalize the fluorescence time courses by the radius of each condensate, we can collapse the fluorescence time course curves (Fig. 1e). The decay portion of the collapsed fluorescence curve appears exponential in time, and we fit this curve to a single-exponential decay model (Fig. 1e and f). The exponential fit timescales are similar across trials in the presence of ATP (Fig. 1g). These results show that RNA leaves the condensate over time with a characteristic timescale that depends on a balance between surface area and the amount of RNA in a condensate.

We next investigated ways to influence LAF-1's helicase activity in order to use these perturbations to study the influence of activity in our reconstituted system. We avoided varying nucleotide concentration as this can have effects on condensate phase behavior through ATP's chemical properties, independent of its identity as a substrate for LAF-1.^42^ Instead, we varied nucleotide substrate identity, which has a less pronounced effect than nucleotide concentration on biomolecular condensation.^42^ To understand how nucleotide identity affects LAF-1's helicase activity, we turned to a bulk fluorescence unwinding assay to study LAF-1's unwinding of a short RNA duplex. The assay monitors the fluorescence of a fluorophore-quencher pair assembled on different strands of an RNA duplex (Fig. S2a). We found that LAF-1 is capable of unwinding a short RNA duplex in the presence of ATP (Fig. S2b and Table S1). The ATP analog ATPγS slows helicase activity and also decreases the total amount of unwound RNA, while LAF-1 is incapable of unwinding the RNA substrate in the presence of ADP (Fig. S2b and Table S1). We note that the finding that LAF-1 is capable of unwinding short stretches of duplexed RNA contradicts the conclusion of a previously published study.^43^ However, it is known that DEAD-box helicase activity is sequence independent but typically inversely related to the strength of a duplex and limited to short stretches of RNA.^24,25^ Therefore, we propose that the previous finding of LAF-1's inability to unwind RNA was a product of the studies’ test substrate (18 base-pairs, 70% GC content) being too strong for LAF-1.^43^ The test substrate used in this study has a melting temperature that is higher than the predicted base-pairing energy of the MS2 hairpin (24 °C *vs.* 22 °C).^44^ From these results, we conclude that LAF-1 can unwind a sufficiently short duplex of RNA and that this unwinding activity is related to its ATPase activity.

We next wanted to assess how impeded LAF-1 helicase activity would influence the time-evolution of RNA gradients we observe in LAF-1:4xMS2 condensates. By impairing LAF-1's helicase activity with ATPγS and ADP, we were able to generate faster time-evolution of the RNA gradients and faster fluorescence decay timescales within the condensed phase (Fig. 1f and Fig. S3). The fit timescale of fluorescence decay also becomes faster as helicase activity is impeded through ATPγS or ADP. (Fig. 1g). This is not due to a change in binding affinity for RNA (Fig. S4 and Table S2). Overall, these results demonstrate that RNA partitioning and homogeneity within the condensed phase is tied to LAF-1's helicase activity. Proper LAF-1 activity promotes RNA incorporation of the 4xMS2 construct and prevents the departure of RNA from the condensed phase.

### Preservation of RNA homogeneity correlates with preservation of RNA dynamics within the condensed phase

**Fig. 2:**
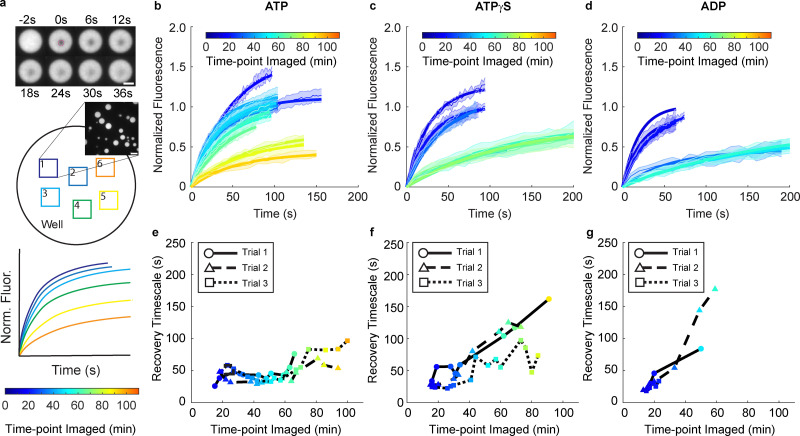
RNA mobility within LAF-1:4xMS2 RNA condensates decreases over time and depends on helicase activity. (a) Fluorescence recovery after photobleaching (FRAP) measurements were performed on 4xMS2 RNA within the condensed phase to measure RNA biomolecular diffusion. Experiments were performed on several condensates within a field of view, and then successive fields of view were measured to account for changes in biomolecular diffusion properties over time. A slowdown in biomolecular diffusion should manifest as slower FRAP recovery. (b)–(d) FRAP time course series of 4xMS2 hairpin RNA in LAF-1:RNA condensates formed in the presence of 1.6 mM (b) ATP, (c) ATPγS, or (d) ADP. Trace color depicts the time-point after system initialization at which the FRAP experiment was performed. Condensates formed in the presence of the different nucleotides initially have similar RNA recovery dynamics. However, a faster slowdown of RNA dynamics is seen as helicase activity is decreased. Error bars depict the standard deviation of the normalized FRAP curves across all droplets in a field of view. Solid lines show single exponential fits to the average normalized FRAP curves. (e)–(g) Recovery timescale as a function of the time at which the FRAP experiment was performed after system initialization for LAF-1:RNA condensates formed in the presence of (e) ATP, (f) ATPγS, or (g) ADP. Data were compiled across three independent realizations. An increase in recovery timescale is seen for condensates formed with ADP and ATPγS after 30–45 minutes of experiment time, with RNA dynamics slowest in the presence of ADP. Recovery amplitude fit parameters are shown in Fig. S5.

Though we found higher helicase activity corresponded to slower 4xMS2 RNA departure from the condensed phase, we did not know whether helicase activity was slowing RNA diffusion to the condensate boundary or instead preventing RNA departure from the condensed phase. To test if the dynamics of the condensed phase were different as a function of unwinding activity, we performed fluorescence recovery after photobleaching (FRAP) on labeled LAF-1 protein and 4xMS2 RNA in the presence of ATP, ATPγS, and ADP. We performed FRAP experiments on different condensates at different points in time to construct time-resolved estimates of biomolecular diffusion as a function of LAF-1 helicase activity (Fig. 2a). RNA recovery dynamics within the condensed phase are initially faster and have similar rates in the presence of ATP, ATPγS, and ADP (Fig. 2b–d). However, over time, the RNA dynamics within the condensed phase slow down or age (Fig. 2b–d). This aging becomes pronounced for LAF-1:4xMS2 RNA condensates formed in the presence of ATP after 60–70 minutes (Fig. 2b and e), while in the case of ATPγS occurs after only 40 minutes (Fig. 2c and f), and in the case of ADP occurs after only 25–30 minutes (Fig. 2d and g). Additionally, the recovery amplitude varies with helicase activity, with higher helicase activity preserving RNA recovery amplitude within the condensed phase for longer (Fig. S5). LAF-1 protein dynamics in the condensed phase were constant over two hours with respect to both timescale and mobile fraction and were insensitive to unwinding activity (Fig. S6). RNA dynamics are therefore tied to higher LAF-1 helicase activity while LAF-1 protein dynamics are not.

That the RNA dynamics within the condensed phase were faster with higher helicase activity suggested to us that the longer RNA departure timescale from the condensed phase with ATP was due to a retention of RNA instead of slower RNA diffusion. Coupled to the faster unwinding activity and larger extent of unwinding with ATP seen in our helicase activity assays, this indicates that the formation of RNA secondary structures may promote RNA departure from the condensed phase. Consistent with this view, if we mix the system such that the RNA can form hairpins before binding with LAF-1 protein, the RNA is excluded from the condensed phase and a pronounced RNA shell forms around the LAF-1 droplets (Fig. S7). This suggests that the timescale we extract in the fluorescence time courses is a timescale related to the RNA folding rate in the system, which strongly depends on helicase activity. With higher LAF-1 helicase activity, there is a lower overall hairpin concentration at any point in time and thus a lower propensity for RNA to leave the LAF-1 condensed phase. With no or lessened LAF-1 helicase activity, hairpins form more quickly and leave the condensed phase.

### An RNA network forms within the condensed phase over time

**Fig. 3:**
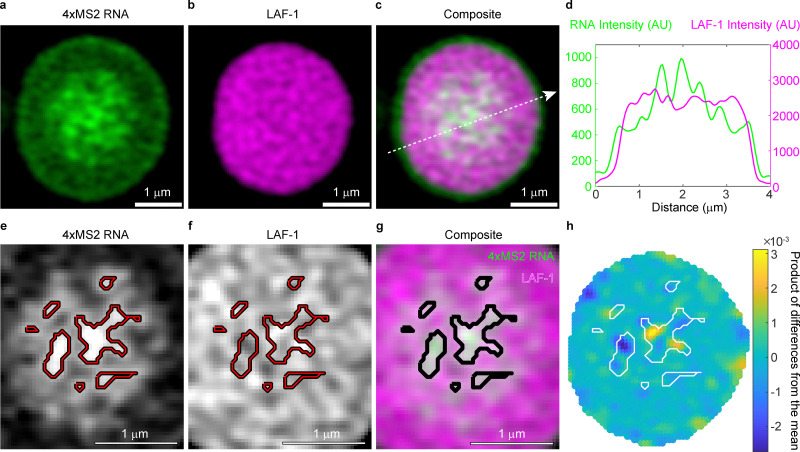
RNA punctate structures form over two hours and correlate with lower LAF-1 fluorescence intensity. (a) 4xMS2 RNA fluorescence, (b) LAF-1 protein fluorescence, and (c) the composite image for a co-condensate formed in the presence of ATP that has aged for 2 hours. Bright RNA puncta are present in the center of the droplet. Scale bar corresponds to 1 µm. (d) Line profile over the 4-micron slice shown in (c), which depicts fluorescence *versus* distance for LAF-1 protein and 4xMS2 RNA. LAF-1 and RNA intensity appear anti-correlated along the line profile, with highest RNA intensity and lowest LAF-1 fluorescence intensity visible at the center of the condensed phase. (e)–(g) Segmenting out the brightest RNA features, these bright RNA regions correlate with local minima in the protein signal. Scale bar corresponds to 1 µm. (h) Normalized product of differences from the mean between the RNA and LAF-1 channels reveal that segmented RNA puncta overlay with negatively correlated regions in the two channels. This suggests these RNA rich regions are locally excluding LAF-1. Refer to Fig. S8 for more examples of this phenomenon.

At the end of the 2-hour time course, we noticed that the initially smooth RNA gradients had turned into punctate structures at the center of the condensed phase, and an RNA shell had emerged at the droplet periphery (Fig. 3a–d and Video S2). The brightest RNA punctate structures at the center of the condensed phase correlate with local minima in the LAF-1 protein signal, suggestive of an RNA network or phase that forms over the course of 2 hours (Fig. 3d–h and Fig. S8). These structures are visible both on a spinning disk confocal microscope and a Zeiss LSM980 laser scanning confocal with an AiryScan 2 detector (Fig. S9). The AiryScan processing does not create the punctate structures as an artifact of processing strength (Fig. S9). Importantly, these punctate structures do not form over time in the absence of the LAF-1 condensed phase, implying they form as a consequence of their high local concentration facilitated by co-condensation with LAF-1 (Fig. S10). These observations are consistent with the recent finding that condensates containing high RNA concentrations can promote RNA aggregation through a percolation transition.^41^ As in our system, this previous study also found that RNA aggregation was associated with a slowdown in RNA dynamics.^41^ The formation of RNA punctate structures which exclude protein along with the slowed RNA dynamics over time suggest the formation of a percolated RNA network and a secondary RNA phase transition that competes with departure of RNA from the condensed phase.

### RNA secondary structure prevents a second phase transition of RNA

**Fig. 4:**
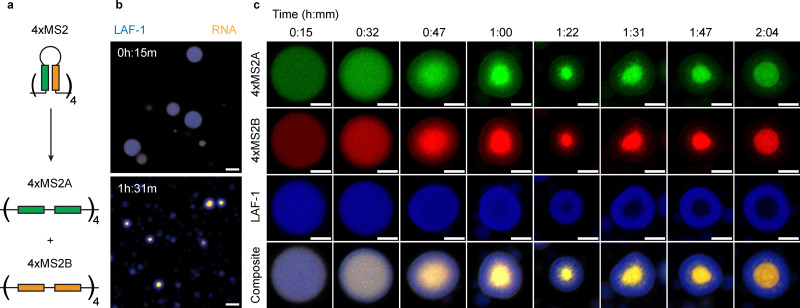
RNA constructs that lack stable RNA secondary structures undergo a second phase transition. (a) The 4xMS2 hairpin construct was split into two separate constructs (4xMS2A and 4xMS2B) that preserve the total base-pairing energy of the MS2 hairpin, but which can only be satisfied intermolecularly. (b) Condensates with both constructs are initially homogeneous in both protein and RNA (Top). After 90 minutes, the RNA in the system condenses at the center of the protein rich phase (Bottom). This second phase transition only occurs for condensates above a critical size. Scale bar corresponds to 5 µm. (c) Snapshots of single condensates of roughly 4 µm diameter over two hours. Complete exclusion of LAF-1 protein from the RNA-rich phase occurs after 80–90 minutes. For a given channel, fluorescence intensity limits were set to be the same across all time points. Scale bar corresponds to 2 µm.

To explore the idea of a competing RNA phase transition at the center of the LAF-1–RNA condensate, we reasoned that preventing RNA from leaving the condensed phase because of hairpin formation would increase the prominence of this event. We split the MS2 hairpin sequence into two separate constructs (4xMS2A, 4xMS2B) which each contain a section of the MS2 base-pairing motif (Fig. 4a and Table S3). We then formed condensates with LAF-1 protein and both 4xMS2A and 4xMS2B RNA in the presence of ATP at the same total RNA mass concentration as in the 4xMS2 experiments. Though both 4xMS2A and 4xMS2B initially incorporate into the LAF-1 condensed phase to form homogeneous protein–RNA condensates, over the course of 90 minutes these constructs undergo a phase transition to an entirely RNA core which excludes LAF-1 (Fig. 4b). This process also seems to depend on condensate size, with no RNA core visible for small droplets (Fig. 4b). A finer time course of this process shows RNA gradients which are initially similar to the complete MS2 hairpin case but without the RNA departure from the condensed phase (Fig. 4c). The RNA concentration at the center of the condensed phase continues to increase until there is a complete phase transition wherein LAF-1 protein is completely excluded (Fig. 4c). This effect does not rely on the specific base-pairing between the MS2A and MS2B motifs (Fig. S11), which suggests it is due to nonspecific, weak base-pairing interactions between the RNA. This then implies the presence of the MS2 hairpin in earlier experiments prevents some of these transient interactions, either by decreasing the number of possible interaction sites or decreasing the conformational entropy of the RNA molecules. This is consistent with previous simulations of RNA condensates, which show an unfolding of RNA secondary structure is a prerequisite for the formation of an RNA-rich phase driven by base-pairing interactions.^35^ The 4xMS2A and 4xMS2B constructs are not capable of forming condensates under identical assay conditions in the absence of LAF-1 protein (Fig. S12), meaning that the presence of the LAF-1 condensed phase is required to promote this secondary RNA phase transition. Lastly, the formation of a second, RNA-rich phase at the center of the condensed phase is not dependent on ATP or ATPase activity, as we observe a similar phenomenon with either ATPγS or ADP (Fig. S13). There is still RNA departure from the condensed phase over time when the system is initialized with ADP (Fig. S13), suggesting weaker RNA structures can still promote RNA's exclusion from the condensed phase. Ultimately, however, this RNA departure is not fast enough to avoid coalescence of a second RNA phase at the center of the LAF-1 condensates (Fig. S13).

## Discussion

**Fig. 5:**
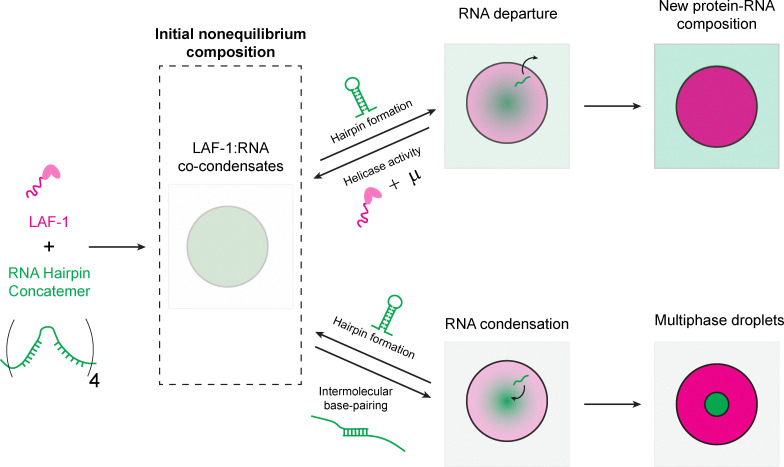
Model: Energy-dependent helicase activity and RNA secondary structure work in concert to preserve an initial nonequilibrium state of condensate homogeneity and dynamics. The formation of 4xMS2 RNA secondary structure promotes RNA exclusion from the condensed phase. LAF-1 RNA helicase activity weakens or prevents MS2 RNA secondary structures from forming, promoting RNA inclusion within the condensed phase. Condensation or aggregation of RNA occurs over time and is mitigated by the presence of RNA secondary structure, which decreases the number of potential RNA–RNA interaction sites, reduces RNA conformational entropy, or both.

In this work, we have demonstrated that the antagonistic interplay between helicase activity and RNA secondary structure can preserve condensate homogeneity, dynamics, and composition. The presence of RNA hairpins facilitates the release of RNA from the condensed phase, while ATP-dependent helicase activity counteracts this by unwinding RNA secondary structures, thereby retaining RNA within the condensate (Fig. 5). Concurrently, RNA hairpins inhibit multiple interactions among RNA molecules, preventing the formation of a separate RNA phase within the LAF-1 protein condensate (Fig. 5). This dynamic tug-of-war ensures condensate homogeneity and promotes effective RNA incorporation, while also preventing the high RNA concentrations within the condensed phase from triggering a secondary phase transition.

These findings have significant implications for the biological role of DEAD-box helicases, particularly in promoting RNA incorporation into biomolecular condensates and preventing excessive local RNA concentrations from leading to aggregation or percolation transitions. Our results also suggest that RNA secondary structures may serve as an additional regulatory mechanism, allowing biological systems to favor RNA departure from the condensed phase when helicase activity alone is insufficient to prevent an RNA phase transition. Moreover, we show that the dynamics of biomolecular condensates containing base-pairing interactions can be actively maintained through energy-dependent helicase activity, which is likely critical for RNA processing and folding pathways localized to these structures in cells.

An important next step emerging from this work is to investigate how LAF-1 DEAD-box helicase activity affects condensate dynamics when longer and more physiologically relevant RNAs are present. Of particular interest are mRNAs found in LAF-1's native condensate, the P granule of *C. elegans*,^45^ RNA found in stress granules,^30,46,47^ and repeat expansion disorder RNA sequences.^37^ Testing the effects of LAF-1 helicase activity in these contexts should help further expand our understanding of the biological functions of DEAD-box helicases and give insight into whether DEAD-box helicase activity's effects on condensates can be leveraged for therapeutic avenues. In considering longer RNAs, the role of RNA tertiary contacts should also become more prominent. The consequences of RNA tertiary structure for biomolecular condensates are poorly understood, though recent examples suggest their importance.^48,49^ For proteins whose interactions with RNA are influenced by RNA tertiary structure, this may be an additional regulatory knob dictating condensate behavior.^50^ For example, DEAD-box helicases bind to RNA upon the transient loss of tertiary contacts,^51^ but RNA binding and consequent RNA unwinding could be hindered by exceptionally stable RNA tertiary contacts. Future studies may study the impact of RNA tertiary structure on DEAD-box helicase condensates through the use of structurally characterized RNAs or through site-specific crosslinking of RNA molecules prior to condensate formation.

Finally, we have developed a novel biopolymer material whose internal enzyme activity maintains its properties in an initial nonequilibrium state. By coupling enzyme activity to the interaction strengths that govern condensate formation and properties, we expand the design space for nonequilibrium condensates, transcending the sequence constraints of their constituent polymers. This work not only deepens our understanding of the fundamental principles governing cellular organization but also opens new avenues for the design of advanced synthetic condensate systems.

## Methods and materials

### LAF-1 expression, purification, and labeling

LAF-1 expression, purification and fluorescent labeling were performed as previously described.^29,32^ Briefly, the LAF-1 coding sequence was inserted into a pET28a(+) vector and was recombinantly expressed in *E. coli* BL21(DE3) cells following induction with 1 mM IPTG. The BL21(DE3) cells were chemically lysed with lysozyme in the presence of Triton X100 and a Roche complete protease inhibitor cocktail tablet, followed by mechanical lysis with a tip sonicator. The lysate was centrifuged and the soluble fraction retained. LAF-1 protein was purified using nickel affinity chromatography followed by heparin affinity chromatography. Purity and yield were assessed *via* SDS-PAGE and absorbance measurements at 280 nm. LAF-1 protein was then dialyzed into buffer containing 10% glycerol, flash frozen in liquid nitrogen, and stored at −80 °C until use.

To generate fluorescently labeled LAF-1 protein, Dylight633-NHS ester (Thermo Scientific 46417) was used following the manufacturer's protocols. Protein was labeled prior to flash freezing.

### *In vitro* transcription and RNA labeling

The DNA sequence for the desired RNA construct was amplified with PCR using a primer that also added a T7 promoter sequence in front of the hairpin sequence. PCR products were run on a 2% agarose gel and gel extracted using the Qiagen QIAQuick gel extraction kit.

*In vitro* transcription was performed using the NEB HiScribe T7 High Yield RNA synthesis kit. RNA sequences for the *in vitro* transcription products used in the presented condensate data are provided in Table S3. For fluorescently labeled RNA, 1 µL of either 10 mM Cy3-UTP (APExBIO) or 10 mM fluorescein-12-UTP (Roche) was included in the 20 µL *in vitro* transcription reaction. RNA was purified *via* lithium chloride precipitation. RNA stocks were diluted to 600 ng µL^−1^ with nuclease free water, flash frozen in liquid nitrogen, and stored at −80 °C for no longer than a month before use.

### Condensate sample preparation

Unlabeled LAF-1 protein and LAF-1 labeled with Dylight-633 was thawed at room temperature before being buffer exchanged into fresh high salt buffer (20 mM Tris pH 7.4, 1 M NaCl, 1 mM DTT). Labeled LAF-1 and unlabeled LAF-1 samples were combined such that the final LAF-1 sample had less than or equal to 1% of the protein labeled. The LAF-1 sample was then diluted to 25 µM in high salt buffer as a working stock for experiments. RNA samples were thawed and stored on ice until use.

Reactions were assembled by mixing 2 µL of RNA at 600 ng µL^−1^ with 1 µL of 3x-folding buffer containing 30 mM Tris pH 7.4 and 30 mM potassium acetate. RNA was heated to 95 °C and then cooled to room temperature in 3 °C/3 minute intervals. The 3 µL RNA sample was then mixed with 3.2 µL of LAF-1 protein at 25 µM in 1 M NaCl, 20 mM Tris pH 7.4, and 1 mM DTT. This mixture was quickly added to another mixture containing 8.8 µL of 20 mM Tris pH 7.4, 1 mM DTT and 1 µL of 25 mM magnesium + nucleotide in 20 mM Tris pH 7.4. The final concentrations of components were then 5 µM LAF-1, 75 ng µL^−1^ of RNA, 1.6 mM magnesium + nucleotide, 20 mM Tris pH 7.4, 200 mM NaCl, and 1 mM DTT.

For the split construct experiments, a 1 : 1 by mass mixture of 4xMS2A and 4xMS2B RNA sample at a total RNA concentration of 600 ng µL^−1^ was prepared. 2 µL of this mixture was then mixed with 1 µL of 3×-folding buffer before proceeding in preparing condensates in the same manner as the 4xMS2 complete construct.

Samples were incubated for 8 minutes before transferring 10 µL to a PDMS chamber adhered to a 1.5H glass coverslip that had been passivated overnight with 2 w/v% Pluronic F-127. The chamber was then mounted on either an Andor Spinning Disk confocal microscope or a Zeiss LSM980 laser scanning confocal microscope.

### Spinning disk confocal fluorescence imaging

Spinning disk fluorescence imaging was performed on a Nikon Ti-E inverted microscope equipped with an Andor iXon 897E EM-CCD camera, a Yokugawa CSU-X1 Spinning Disk Confocal Scan Head. A 100× Nikon objective with a numerical aperture of 1.4 was used for all spinning disk imaging.

### Zeiss Airyscan 2 fluorescence imaging

Laser scanning confocal imaging was performed on a Zeiss LSM980 laser scanning confocal equipped with a 63×, 1.4 NA, oil objective and an Airyscan 2 detector. Airyscan processing was done with default processing settings. Image grids of 3 × 3 (Big Image) were used for imaging to increase droplet imaging throughput without producing long time-delays in vertical slices. Imaging intervals of 5 minutes between Big Images were used for all datasets. Imaging was done at 21–22 °C.

In analyzing the time courses, individual condensates were segmented and tracked in MATLAB using elements of the Image Processing Toolbox. Information about condensate fluorescence and shape could then be extracted using custom MATLAB scripts.

Radial profiles were generated in 2-dimensions by averaging pixel intensities at rings with increasing radius from the identified condensate center. Radial profiles were constructed from condensates whose midplanes had an average circularity of at least 0.9 and averaged radial profiles were computed using a radial bin size of 0.24 µm.

To measure the intensity at the center of each condensate, the midplane of each condensate was identified by finding the *z*-slice with the maximum number of pixels. The centroid of this midplane was then calculated and the average pixel intensity within one pixel of the centroid was calculated. Intensity values for the same condensate at different points in time were linked *via* trajectory construction performed on the midplane centroids. Intensity as a function of time curves were fit to single exponential decay models using Nonlinear Least Squares Curve Fitting in MATLAB.

For colocalization analysis, droplets were first segmented relative to the protein channel. A mask was applied using the protein contour, followed by a second segmentation based on the RNA channel. This second segmentation procedure was used to identify RNA puncta in the condensed phase. Colocalization between RNA and protein signal within the droplet contour was calculated using a normalized product of differences from the mean:
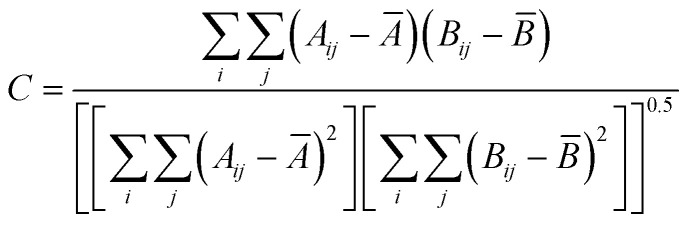
where *i* and *j* denote row and column of the images, *A* denotes Channel 1 and *B* denotes Channel 2 of the image, and *Ā*, *B̄* are the averages of the Channel 1 and Channel 2 images, respectively.

### Fluorescence recovery after photobleaching

Fluorescence imaging was performed on a Nikon Ti-E inverted microscope equipped with an Andor iXon 897E EM-CCD camera, a Yokugawa CSU-X1 Spinning Disk Confocal Scan Head, and an Andor FRAPPA photomanipulation system. A 100× Nikon objective with a numerical aperture of 1.4 was used for all FRAP experiments. For FRAP experiments, a spot size of 3 pixels, corresponding to roughly 0.42 µm, was used for photobleaching. FRAP time courses were generated by FRAPping several droplets within a field of view to get statistics on recovery timescale at a particular time-point. Different fields of view were then imaged at successive time-points to generate information about recovery timescale as a function of the time at which the experiment was performed.

Individual droplets which had FRAP regions were cropped and bleach spots were identified using custom Python scripts (https://github.com/stcoupe/FRAP-analysis). Intensity within the bleached region was normalized relative to the intensity outside the bleach spot but within the droplet. We then performed an affine transformation of the data in order to be able to directly compare FRAP curves. The intensity ratio at the first bleach time-point (*I*(0)) was subtracted from each time-point (*I*(*t*)), and this was then divided by the difference between the average intensity ratio before bleaching (*I*_*i*_) and *I*(0).
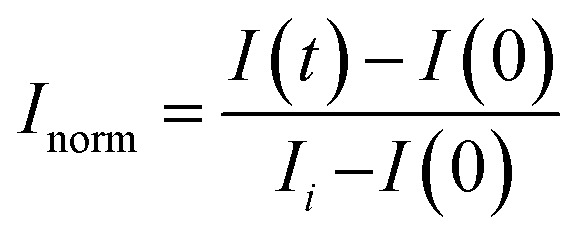
Note that this is a different normalization procedure than typical for condensate measurements.^52^ However given the gradients present particularly in the RNA channels of our condensed phases, the modified normalization procedure was used.

Normalized FRAP curves for all droplets in a field of view were averaged together. The average normalized FRAP curve was then fit to a single exponential using MATLAB's fit function to measure the FRAP recovery timescale and amplitude for a given timepoint following droplet formation.

## Author contributions

S. C. conducted all experiments and analysis of experimental data. S. C. and N. F. designed the research, interpreted the results, and co-wrote the manuscript.

## Conflicts of interest

There are no conflicts to declare.

## Supplementary Material

SM-022-D5SM01106J-s001

SM-022-D5SM01106J-s002

SM-022-D5SM01106J-s003

## Data Availability

The Python code used to generate FRAP time courses is available at: https://github.com/stcoupe/FRAP-analysis. The MATLAB code used to analyze fluorescence time courses is available at: https://github.com/stcoupe/LAF-1_Hairpin_Analysis/. The raw data associated with this study, including fluorescence time courses, FRAP time courses, fluorescence colocalization data, fluorescence polarization data, and real-time fluorescence unwinding data, can be obtained from the corresponding author upon reasonable request. References cited in the supplementary information (SI) have been included at the end of the references section in the main text. Supplementary information: ref. 25, 53 and 54 are cited. See DOI: https://doi.org/10.1039/d5sm01106j.
