# Supplementary material for: Nonequilibrium phases of a biomolecular condensate facilitated by enzyme activity
Source: Soft Matter. 2026 Mar 12;22(14):2656–65. doi: 10.1039/d5sm01106j (PMC12999270; doi:10.1039/d5sm01106j)
Supplement: SM-022-D5SM01106J-s001 [file SM-022-D5SM01106J-s001.pdf]

Supplementary Information (SI) for Soft Matter.
This journal is © The Royal Society of Chemistry 2026

Supplementary Information

Nonequilibrium phases of a biomolecular condensate facilitated by enzyme activity

Sebastian Coupe[1,2,3], Nikta Fakhri[2,4]

[1] Department of Biology, Massachusetts Institute of Technology, Cambridge, MA, USA
[2] Department of Physics, Massachusetts Institute of Technology, Cambridge, MA, USA
[3] Bioengineering Department, Stanford University, Stanford, CA, USA
[4] Corresponding author: fakhri@mit.edu

1 Extended Methods
1.1 Unwinding Assays . . . .
1.2 Hairpin Construct Cloning . . . .
1.3 Fluorescence Polarization . . . .

2 Supplementary Videos

3 Supplementary Tables

4 Supplementary Figures

1 Extended Methods

1.1 Unwinding Assays

Unwinding assays were designed and conducted as previously described [52]. RNA and DNA oligonucleotides were ordered from IDT as described below. Oligonucleotides were prepared as 100 μM stocks in nuclease free water.

Sequences of oligonucleotides used in the unwinding assay:
Fluorescent Strand: 5'-Alexa488-rArGrUrArGrGrUrC-3'
Quencher Strand: 5'-$(rU)_{20}$-rGrArCrCrUrArCrU-IowaBlackFQ-3'
Competitor DNA: 5'-GACCTACT-3'

RNA duplexes were prepared by annealing 200 μL of 1μM fluorescent RNA strand with 20 μL quencher RNA at 10 μM in 180 μL of reaction buffer (20 mM Tris pH 7.4, 2 mM magnesium acetate, 0.2 mM DTT). The reaction was heated to 80°C in a thermocycler and then cooled to 21°C in 3°C/3-minute increments. The reaction was spun down at 10,000xg for 10 seconds, then placed on ice for 5 minutes. 250 μL of the annealing mixture was then transferred to a fresh 1.7 mL microcentrifuge tube, along with 12.5 μL of 100 μM competitor DNA and 237.5 μL of 1x reaction buffer to produce a final duplex stock concentration of 250 nM.

LAF-1 protein was thawed at room temperature and buffer exchanged into fresh high salt buffer (20mM Tris pH 7.4, 1M NaCl, and 1 mM DTT). Protein 10x stock solution was then prepared at 5 μM in 20mM Tris pH 7.4, 1M NaCl, and 1mM DTT.

Fluorescence measurements were taken on a Tecan Infinite M200 Pro with fluorescence capabilities using an excitation filter of 488 nm with a bandwidth of 9 nm and an emission filter of 530nm with an emission bandwidth of 20nm. The gain was set at 125 and 5 flashes were used for the measurement, each with an integration time of 1 msec.

A maximum intensity calibration sample was prepared consisting of 50 nM fluorescent RNA, 500 nM DNA competitor, 500 nM LAF-1, 2.5 mM MgATP in reaction buffer. 70 μL of sample was transferred to a 96-well all-black fluorescent plate. Fluorescence readings were taken at 1 second intervals until a plateau in signal was reached, which was used as the maximum unwinding signal. Maximum calibration measurements were performed in triplicate.

Reaction mixtures were prepared with 50 nM annealed RNA and 500 nM LAF-1 in 1x reaction buffer. 63 μL of the mixture was transferred to the fluorescent plate and fluorescence readings were taken until a plateau was reached. At this point, 7 μL of 25 mM MgATP, MgATPγS, or MgADP was added to the mixture, the sample was mixed quickly, and then fluorescence readings were taken of the unwinding reaction. Reactions were performed in triplicate.

Fluorescence unwinding curves were normalized by first subtracting timecourse values from their reading at time = 0s. They were then divided by the maximum fluorescence calibration reading, less the intensity at time = 0s. Normalized curves were fit using the fit function in MATLAB. Two fit types were performed, a linear model of the initial rates and a single exponential fit. For the linear fit, an R^2 value and RMSE were computed. For the exponential fit, 95% confidence intervals are reported for each fitting parameter (Table S1).
Initial rates:

$$F_{unwound} = k \times t + intercept$$

Single Exponential:

$$F_{unwound} = A(1 - e^{-bt})$$

1.2 Hairpin Construct Cloning

The pSL vector used for hairpin cloning was derived from the pSL-MS2-12x Addgene construct. pSL-MS2-12X was a gift from Robert Singer (Addgene plasmid # 27119 ; `http://n2t.net/addgene:27119` ; RRID:Addgene_27119) [1].

The DNA sequences for single hairpins were ordered from IDT. They were then inserted into a pSL vector with a 5'-BamHI restriction site and a 3'-BglII restriction site. To create concatenated hairpins, we followed a procedure similar to the one used to originally clone the 12xMS2 hairpin construct [1]. A shorter concatemer number than 12x was desired as this significantly increased in vitro transcription yield while enabling study of base-pairing interactions. We first amplified the single hairpin using polymerase chain reaction (PCR), followed by restriction digest and gel purification. The DNA sequence for the single hairpin was then ligated with T4 ligase but in the presence of BamHI and BglII, in NEB buffer 3.1 with 1mM additional ATP. The BamHI and BglII sites have one nucleotide of overhang that is complementary. In this way, we achieved successful head-to-tail ligation which the restriction enzymes cannot cleave when the overhangs ligated in the BamHI-BglII site configuration. Reactions were allowed to proceed overnight at 37°C.

After overnight incubation, the samples were run on a 2% agarose gel and gel purified. PCR was then run on the samples to both increase concentration of the concatemers and introduce Gibson assembly overhangs on the concatemers. A second agarose gel and gel extraction step was performed on the products of the Gibson PCR. Many different concatemers were present at this step, and bands corresponding to the desired repeat number were selected. Gibson assembly was performed on the selected hairpin repeat constructs with the pSL vector backbone followed by a 4x dilution with water and transformation into NEB5α competent cells. Single colonies were picked and grown in LB media before miniprepping and Sanger sequencing.

1.3 Fluorescence Polarization

An RNA oligo of rU20 with a 5'-Alexa488 fluorophore was synthesized by IDT.

LAF-1 protein was thawed at room temperature before being buffer exchanged into fresh high salt buffer (20 mM Tris pH 7.4, 1 M NaCl, 1 mM DTT). Protein samples were then diluted to 4 μM with high salt buffer and then concentration measurements were then taken in triplicate using a Tecan M200 plate reader with the Nanoquant insert. The samples were then diluted 5-fold with low salt buffer (20 mM Tris pH 7.4, 1 mM DTT) to produce the stock protein concentrations for the serial dilution.

Serial dilutions of 1.63x were prepared by filling the wells of a Corning 96-well flat bottom black plate with 105 μL of assay buffer (20 mM Tris pH 7.4, 200 mM NaCl, 1 mM DTT) containing 2 nM fluorescent RNA oligo and the desired concentration of nucleotide. 180 μL of protein stock solution containing 2 nM fluorescent RNA oligonucleotide and the desired concentration of nucleotide was then added to the first well, and successive volumes of 180 μL were transferred to subsequent wells. Samples were incubated for 5 minutes before fluorescence polarization readings were taken. Incubation time was tested to ensure the system had equilibrated by this

point. Experiments were performed in triplicate.

Fluorescence polarization measurements were performed with a Tecan Infinite M1000Pro, using a 470nm excitation filter with a 5 nm bandwidth, a 520nm emission filter with a 10 nm bandwidth, 110 gain, and 10 flashes per reading. The G-factor for the fluorescence polarization was calibrated using fluorescein, and determined to be 1.162.

Fluorescence polarization as a function of protein concentration was plotted and fit to a Hill equation using Nonlinear Least Squares Fitting performed in MATLAB. A vertical offset (b) and amplitude term (A) were included to fit the data:

$$FP = A\frac{[LAF1]^n}{([LAF1]^n + K_D^n)} + b$$

Fit parameters and their 95% confidence intervals can be found in Table S2.

References

1. Bertrand, E. *et al.* Localization of ASH1 mRNA Particles in Living Yeast. *Molecular Cell* **2,** 437–445. ISSN: 1097-2765. `https://www.sciencedirect.com/science/article/pii/S1097276500801434` (1998).
2. Özes, A. R., Feoktistova, K., Avanzino, B. C., Baldwin, E. P. & Fraser, C. S. Real-time fluorescence assays to monitor duplex unwinding and ATPase activities of helicases. *Nature Protocols* **9,** 1645–1661. `https://doi.org/10.1038/nprot.2014.112` (June 2014).
3. Linder, P. & Jankowsky, E. From unwinding to clamping – the DEAD box RNA helicase family. *Nature Reviews Molecular Cell Biology* **12,** 505–516. ISSN: 1471-0080. `https://doi.org/10.1038/nrm3154` (2011).

2 Supplementary Videos

Video 1: LAF-1:4xMS2 condensates exhibit time-dependent RNA gradients and composition changes. Two-hour fluorescence timecourse of LAF-1:4xMS2 RNA condensates formed in the presence of 1.6 mM MgATP. RNA gradients emerge over time and RNA gradually leaves the condensed phase. 4xMS2 hairpin RNA is labelled with fluorescein-UTP (LEFT) while LAF-1 protein is labelled with Dylight-633 (MIDDLE). Their composite image is depicted on the right (RIGHT). Scale bar is equal to 5 microns, the timescale shown is in hh:mm.

Video 2: An RNA network forms at the center of LAF-1:4xMS2 condensates over the course of two hours. Top Row) A midplane image of a LAF-1:4xMS2 co-condensate formed in the presence of 1.6 mM ATP and aged for 2 hours at room temperature. 4xMS2 RNA (LEFT), LAF-1 protein (MIDDLE), and their composite image (RIGHT). Scale bar is equal to 0.5 microns. Bottom Row) A rotating video of the 3D volume for the same LAF-1:4xMS2 co-condensate. An enriched RNA core is seen at the center of the condensed phase, where this core possesses a coarse, aggregate-like structure. A slight depletion in LAF-1 protein signal can also be seen at the center of the condensate.

3 Supplementary Tables

Condition	$k_{initial\ rates}$ (fraction/s)	Intercept (fraction)	Linear R^2	Exp. A (fraction)	$k_{exponential}$ (s^{-1})
ATP	0.0134	-0.0186	0.996	0.777 (0.763, 0.790)	0.0246 (0.0232, 0.0259)
ATPγS	0.00224	-0.0162	0.983	0.289 (0.286, 0.291)	0.0107 (0.0103, 0.0111)
ADP	-0.00023	-0.00354	0.724	-0.0179 (-0.0186, -0.0173)	0.0354 (0.0239, 0.0470)

Table 1: Kinetic parameters for LAF-1 duplex unwinding assays performed with different nucleotides. Parameters were extracted from either a linear fit to the initial unwinding rates (columns 2-4) or single exponentials of the entire curve (columns 5 and 6). R^2 values report on the goodness of fit for the linear fit parameters in columns 2 and 3. For the exponential fits, 95% confidence intervals of fit parameters are displayed in parentheses.

Condition	$K_{D,\ App}$ (nM)	$K_{D,\ App}$, 95% CI (nM)	n	n, 95% CI
Buffer	25.2	(23.1,27.2)	1.68	(1.48,1.88)
$MgCl_2$	22.6	(20.3, 24.8)	1.95	(1.62,2.28)
MgATP	11.2	(9.44,13.0)	0.85	(0.73,0.96)
MgATPγS	14.6	(6.5, 22.7)	1	-
MgADP	8.53	(5.06,12.0)	0.54	(0.39,0.70)

Table 2: Apparent binding affinity parameters for LAF-1 fluorescence polarization assays in the presence of different nucleotides and their 95% confidence intervals. Fluorescence polarization curves in Figure S4 were fit to a Hill model where n was included as a fit parameter except in the case of ATPγS where it was fixed at 1.

Construct	Transcribed RNA Sequence (5’ to 3’)
4xMS2	gggacgucgaccugagguaauuauaacccgggcccuauauauggauccuaagguaccuaauu<br>gccuacuagaaaacaugaggaucacccaugucugcaguauucccggguucauuagauccuaa<br>gguaccuaauugccuacuagaaaacaugaggaucacccaugucugcaguauucccggguuca<br>uuagauccuaagguaccuaauugccuacuagaaaacaugaggaucacccaugucugcaguau<br>ucccggguucauuagauccuaagguaccuaauugccuacuagaaaacaugaggaucacccau<br>gucugcaguauucccggguucauuagaucugcgcgcgaucgauaucagcgcuuuaaauuugc<br>gcaugcuccgcugagcaauaacuagc
4xMS2A	gggacgucgaccugagguaauuauaacccgggcccuauauauggauccuaagguaccuaauu<br>gccuacuagaaaacaugaggaucaacaugaggcugcaguauucccggguucauuagauccua<br>agguaccuaauugccuacuagaaaacaugaggaucaacaugaggcugcaguauucccggguu<br>cauuagauccuaagguaccuaauugccuacuagaaaacaugaggaucaacaugaggcugcag<br>uauucccggguucauuagauccuaagguaccuaauugccuacuagaaaacaugaggaucaac<br>augaggcugcaguauucccggguucauuagaucugcgcgcgaucgauaucagcgcuuuaaau<br>uugcgcaugcuccgcugagcaauaacuagc
4xMS2B	gggacgucgaccugagguaauuauaacccgggcccuauauauggauccuaagguaccuaauu<br>gccuacuagaaacccauguaucacccaugucugcaguauucccggguucauuagauccuaag<br>guaccuaauugccuacuagaaacccauguaucacccaugucugcaguauucccggguucauu<br>agauccuaagguaccuaauugccuacuagaaacccauguaucacccaugucugcaguauucc<br>cggguucauuagauccuaagguaccuaauugccuacuagaaacccauguaucacccaugucu<br>gcaguauucccggguucauuagaucugcgcgcgaucgauaucagcgcuuuaaauuugcgcau<br>gcuccgcugagcaauaacuagc

Table 3: Transcribed RNA sequences for RNA constructs used in condensate assays.

4 Supplementary Figures

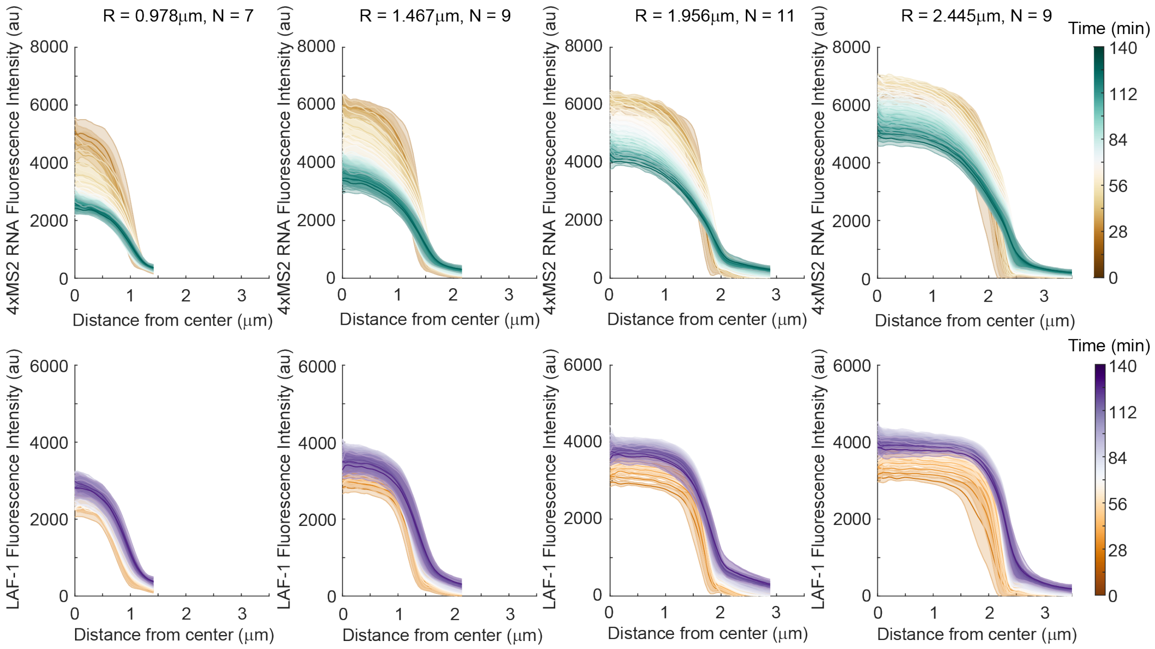

Figure S1: Average radial fluorescence intensity profiles for circular droplets of various sizes. Number of droplets included in the average and the radial bin center are indicated at the top. Each bin has a width of 0.245 μm. Top Row) Average 4xMS2 RNA radial intensity profiles as a function of time. Gradients emerge more quickly and RNA fluorescence decreases faster in condensates of smaller sizes. This is consistent with RNA leaving from the condensate boundary. Bottom Row) LAF-1 radial intensity profile as a function of time. Protein profiles spatially match those of RNA at early times. No gradients are present in the LAF-1 profiles until the condensate boundary is encountered. Protein fluorescence increases slightly over time.

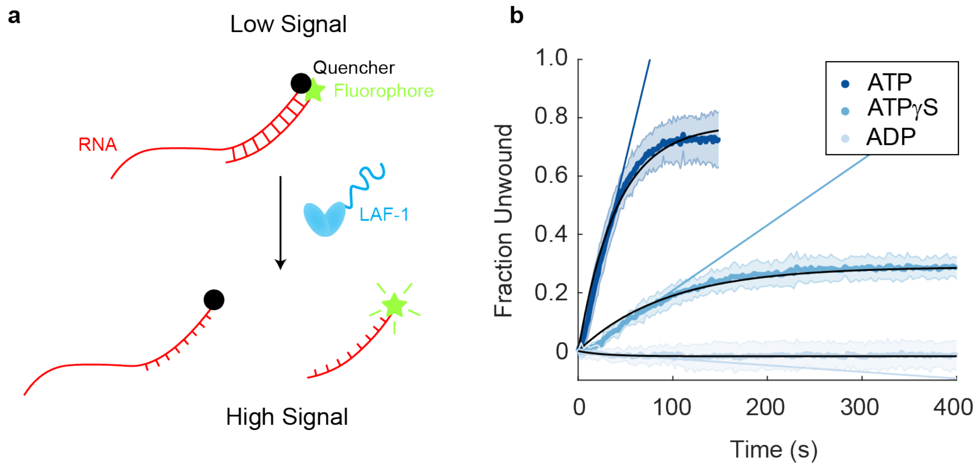

Figure S2: LAF-1 can unwind a short RNA duplex in an ATP-dependent manner. a) Overview of the real-time fluorescence-based unwinding assay [2]. Two RNA strands are annealed such that a fluorophore-quencher pair are in close proximity in the duplexed configuration. One of the RNAs contains a ssRNA overhang, known to be important for other DEAD-box helicases' unwinding activities [3]. Upon unwinding, the fluorophore is liberated and the sample increases in fluorescence emission. b) Normalized unwinding curves from an unwinding assay performed on an RNA construct with a 50% GC content, 8-bp duplex and a 5'-U_{20} ssRNA overhang. Assays were performed in the presence of 500nM LAF-1, 100 mM NaCl, and 2.5mM of the indicated nucleotide. Fastest unwinding rate and highest unwinding fraction are seen with the system containing ATP. ATPγS results in slower unwinding and a lower overall fraction of RNA unwound. ADP does not enable RNA unwinding by LAF-1. Fits were performed to single exponentials (solid black lines) and linear fits of the initial unwinding rates (solid colored lines) (Table S1). Error bars are the standard deviation over four independent replicates for ATP or three independent replicates for ATPγS and ADP.

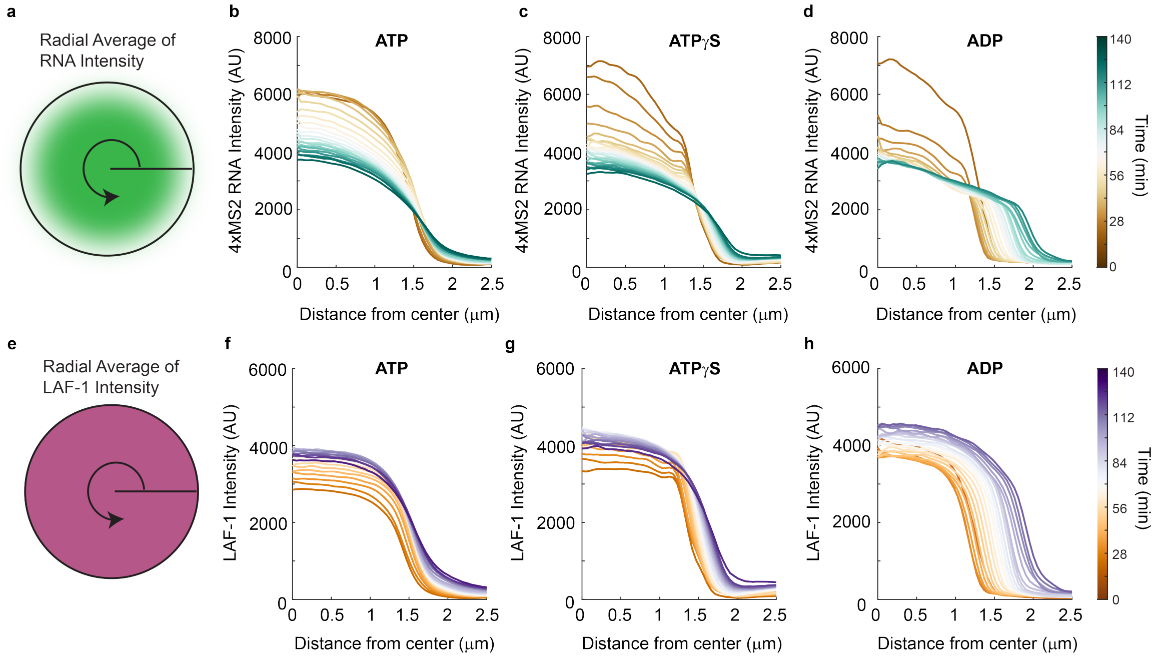

Figure S3: Radial gradient time-evolution depends on nucleotide identity. a) Average radial RNA intensity profile for LAF-1:4xMS2 RNA co-condensates formed in the presence of (b) ATP, (c) ATPγS, or (d) ADP. A faster evolution of RNA gradients and RNA fluorescence decay are seen as helicase activity is impeded with ATPγS and abrogated with ADP. e) Average radial LAF-1 protein intensity profile for LAF-1:4xMS2 RNA co-condensates formed in the presence of (f) ATP, (g) ATPγS, or (h) ADP. Protein intensity increases in the condensed phase with ATP, ATPγS, and ADP, with no spatial variation of protein signal within the condensed phase, and an increase in signal over time. Number of droplets included in each average: (b,f) 9 for ATP, (c,g) 9 for ATPγS, (d,h) 6 for ADP. All droplets averaged had a radius of 1.71 μm +/- 0.122 μm and an average circularity of > 0.9.

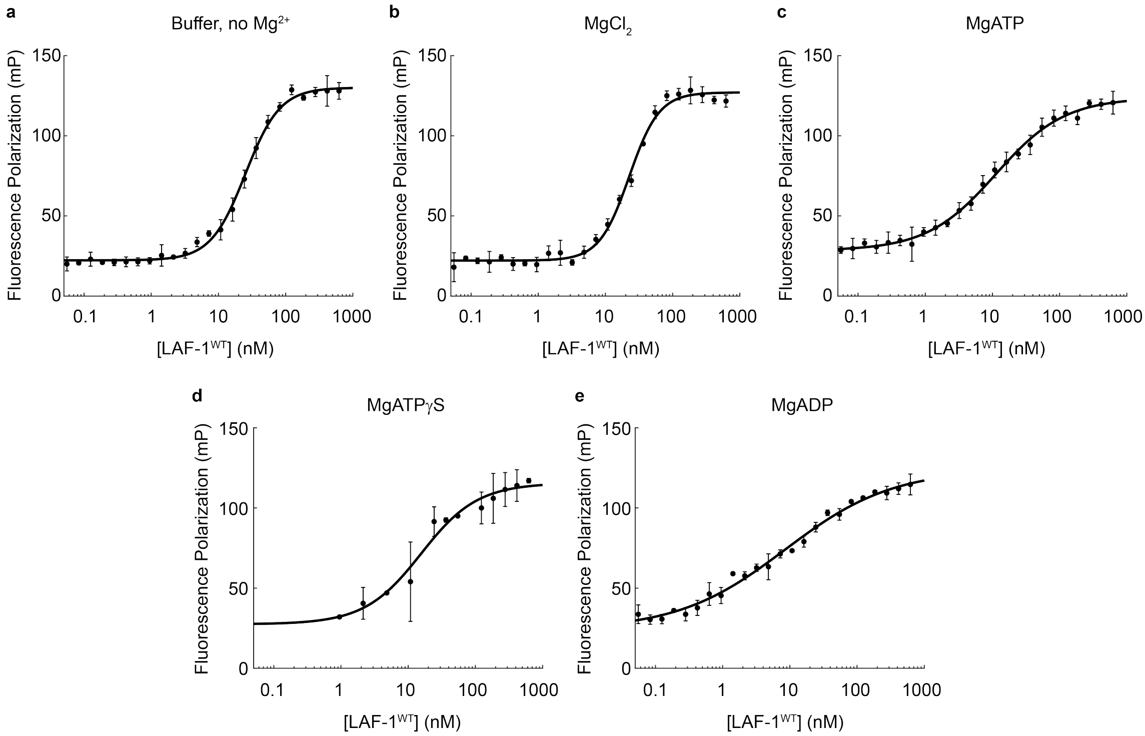

Figure S4: LAF-1 binds RNA with the same apparent affinity in the presence of a variety of nucleotides, but nucleotide presence decreases cooperativity of RNA binding. (a-e) Fluorescence polarization curves comparing LAF-1 binding to rU_{20} in (a) buffer, (b) buffer + 1.6mM $MgCl_2$, (c) 1.6 mM MgATP, (d) 1.6 mM MgATPγS, or (e) 1.6 mM MgADP. Error bars are the standard deviation over three independent replicates. Solid line represents the fit of the data to a Hill binding model, where cooperativity or n was included as a fit parameter, except for ATPγS where n was fixed at 1. The apparent dissociation constants, Hill coefficients, and 95% confidence intervals for the fit parameters are displayed in Table S2.

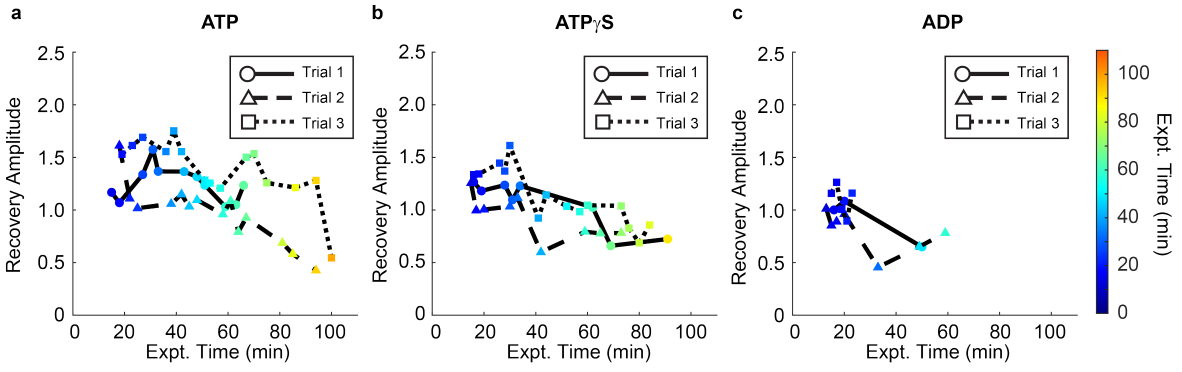

Figure S5: Hairpin RNA mobility within LAF-1 condensates decreases over time and depends on helicase activity. a-c) FRAP recovery amplitudes from the FRAP series of 4xMS2 hairpin RNA in LAF-1:RNA condensates shown in Figure 2. Condensates were formed in the presence of 1.6mM (a) ATP, (b) ATPγS, or (c) ADP. A faster drop in recovery amplitude occurs as helicase activity decreases. Recovery amplitudes greater than 1 are a result of RNA gradients in the condensed phase, with higher RNA concentrations at the center of the droplet.

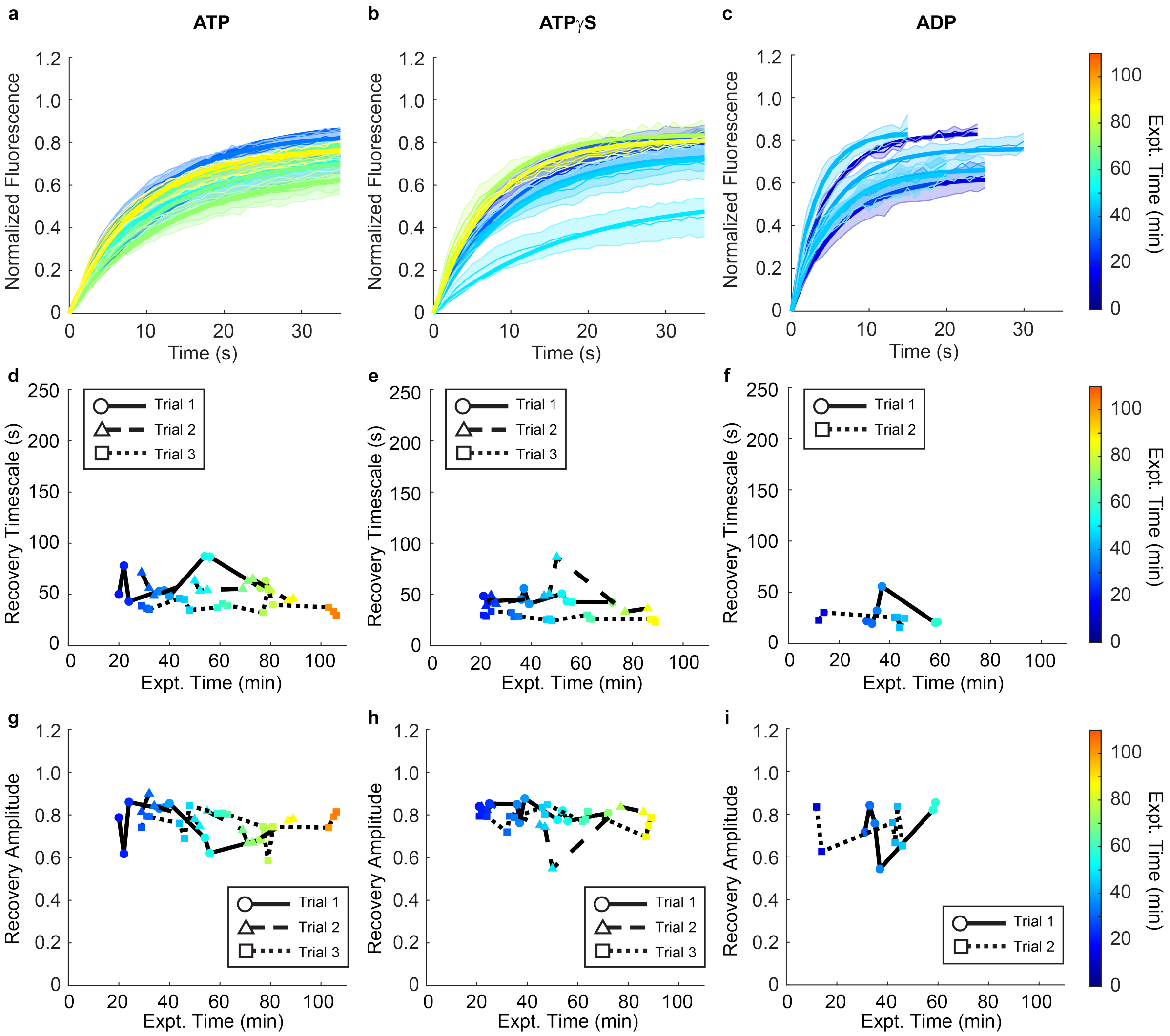

Figure S6: LAF-1 protein mobility in LAF-1:4xMS2 hairpin condensates does not change over time and is independent of helicase activity. a-c) FRAP timecourse series of LAF-1 protein in LAF-1:4xMS2 RNA condensates formed in the presence of 1.6mM (a) ATP, (b) ATPγS, or (c) ADP. There is no difference in LAF-1 protein dynamics with different nucleotides present and LAF-1 protein dynamics do not change over time. d-f) Recovery timescale and (g-i) recovery amplitude as a function of the time at which the FRAP experiment was performed after system initialization for the indicated nucleotides. No difference in protein dynamics is seen between systems with different nucleotides, nor is there time-evolution of protein dynamics.

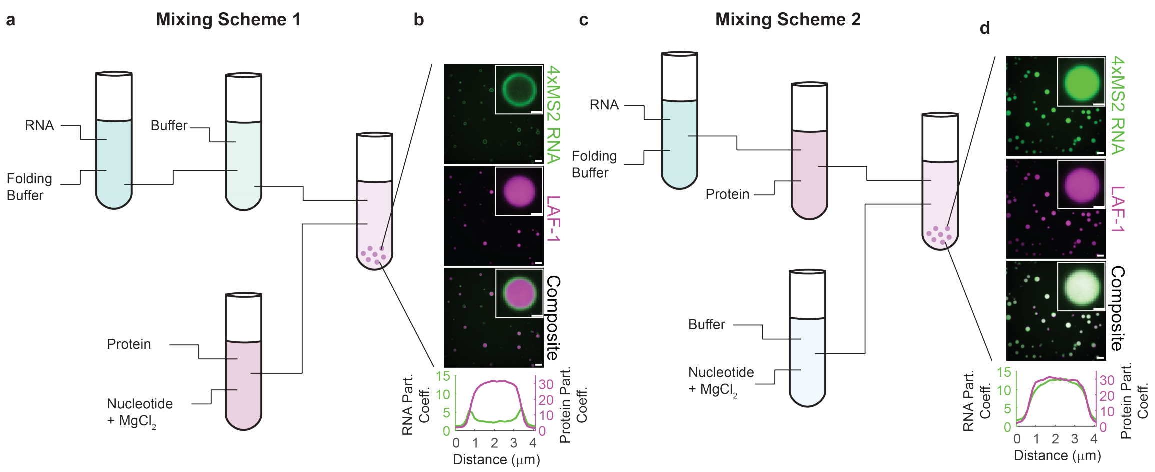

Figure S7: System mixing order affects droplet morphology. a) In the first reaction scheme, 4xMS2 RNA is annealed in buffer containing 10 mM Tris pH 7.4 and 10 mM KAc by heating to 95°C and then cooling to room temperature. This RNA mixture is added to low salt buffer, and a mixture of LAF-1 protein in high salt buffer with nucleotide and magnesium are then added to initiate condensate formation. b) This reaction scheme results in core-shell structures of the droplets, where RNA accumulates at the LAF-1 condensate surface. We believe this is due to RNA secondary structure formation prior to condensate formation. Scale bar of larger micrograph $= 5\ \mu$m, inset scale bar $= 1\ \mu$m. c) In the second reaction scheme, 4xMS2 RNA is annealed as described above, but first added to the LAF-1 protein in high salt buffer. This protein-RNA mixture is then added to low salt buffer containing ATP and magnesium to initiate condensate formation. d) This second reaction scheme produces initially homogeneous LAF-1:RNA co-condensates. We believe this to be due to condensate formation and RNA inclusion in the condensed phase prior to stable RNA secondary structure formation. Scale bar of larger micrograph $= 5\ \mu$m, inset scale bar $= 1\ \mu$m.

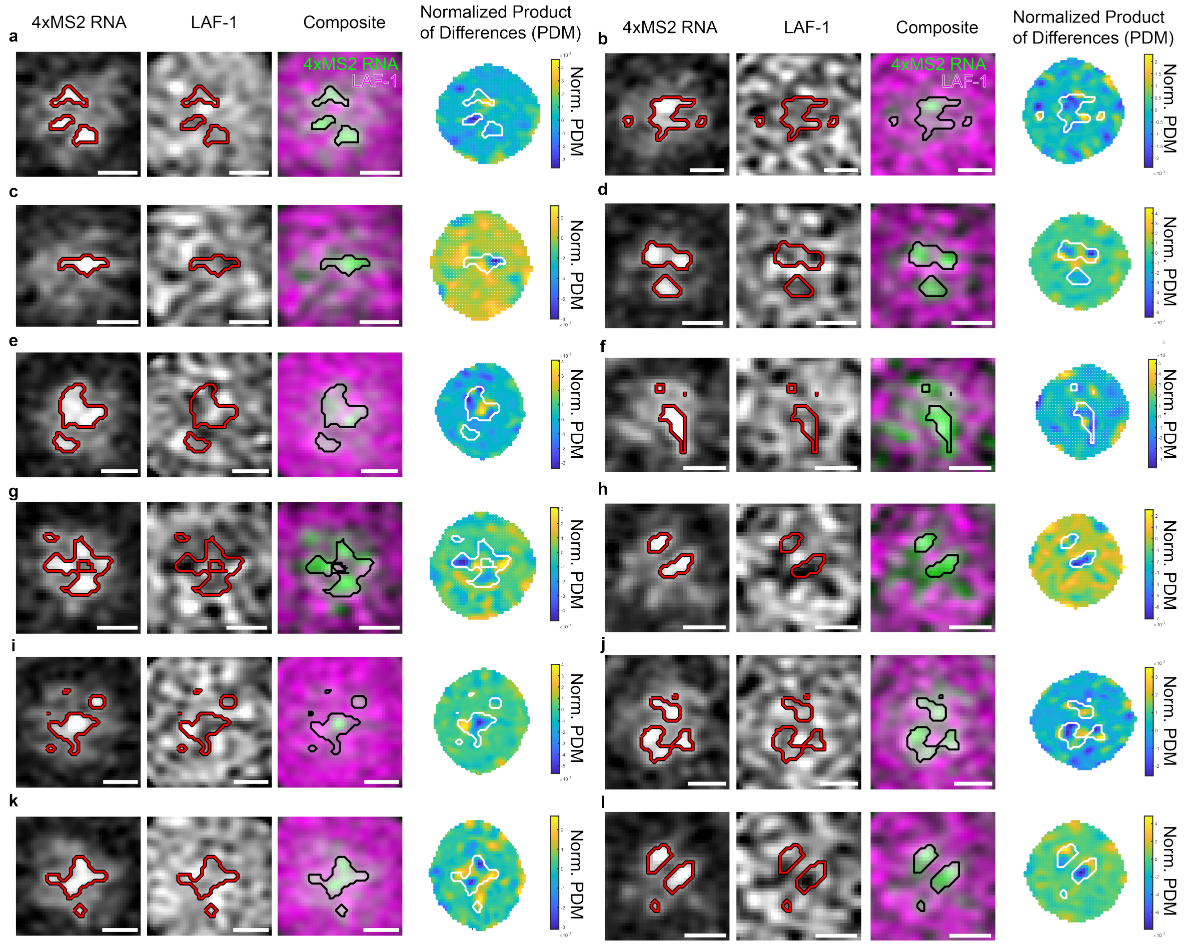

Figure S8: Normalized product of differences from the mean (PDM) analysis of different condensates. Additional examples of RNA punctate structures excluding LAF-1 protein from separate condensates. A more negative PDM value (more blue) indicates stronger anti-correlation between protein and RNA channels. Scale bar = 0.51 μm. Related to Figure 3.

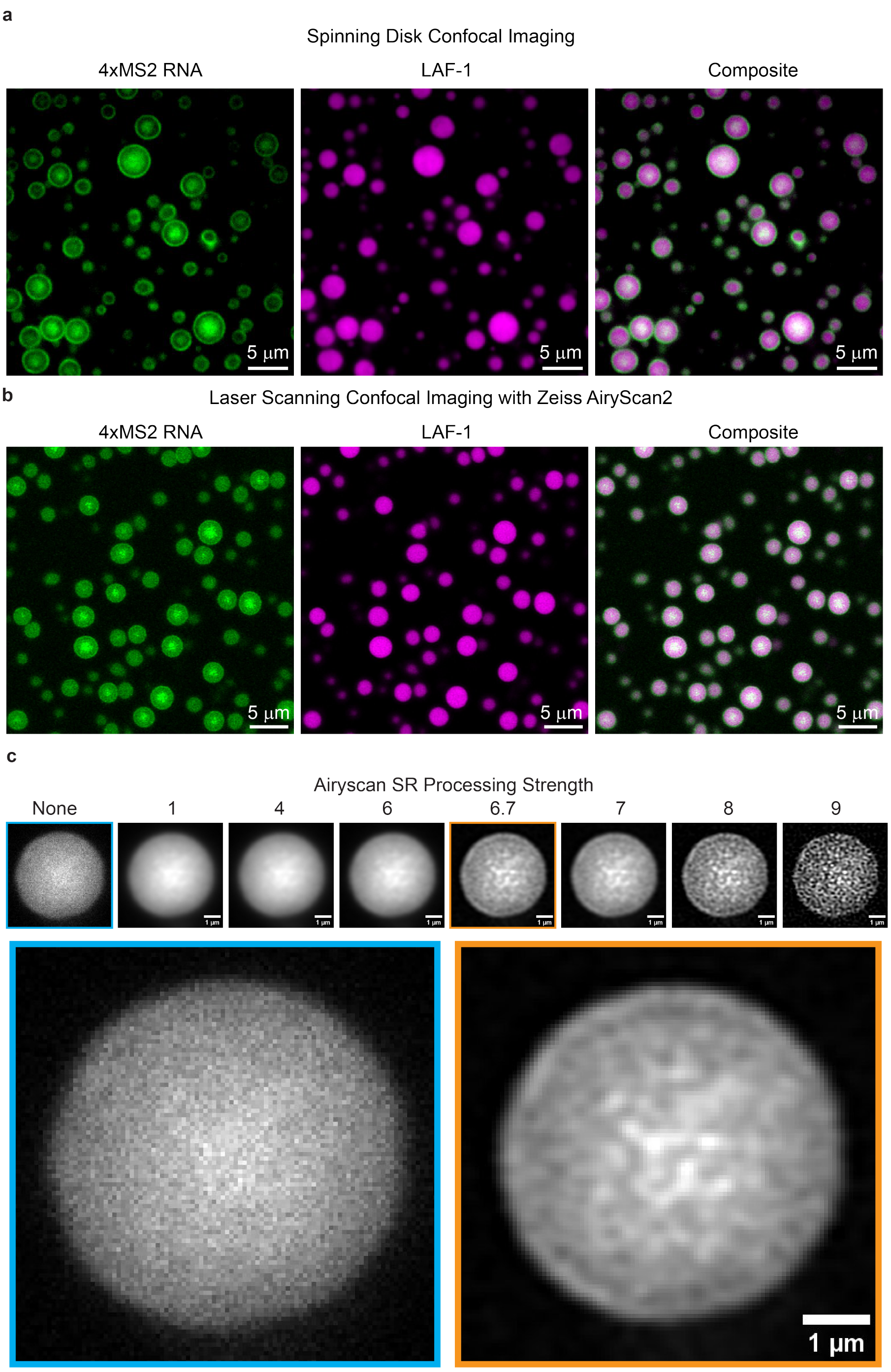

Figure S9: A coarse RNA structure is visible at the interior of LAF-1:4xMS2 RNA condensates after 2 hours. a) Imaging performed on a spinning disk confocal microscope shows coarse RNA structures at the core of LAF-1:4xMS2 condensates but with low spatial resolution. Scale bar = 5μm. b) Imaging with a laser scanning confocal equipped with an Airyscan 2 detector increases spatial resolution of condensate imaging and shows RNA puncta at the core of LAF-1-RNA condensates. Scale bar = 5μm. c) AiryScan processing strength changes the clarity of the spatial structure at the center of the condensate without introducing artificial spatial structure. Left to right shows the effect of increasing Airyscan processing strength. Shown below are larger images of the no Airyscan processing version (Left) and the image processed with the automatically detected Airyscan processing strength of 6.7 (Right). Scale bar = 1μm.

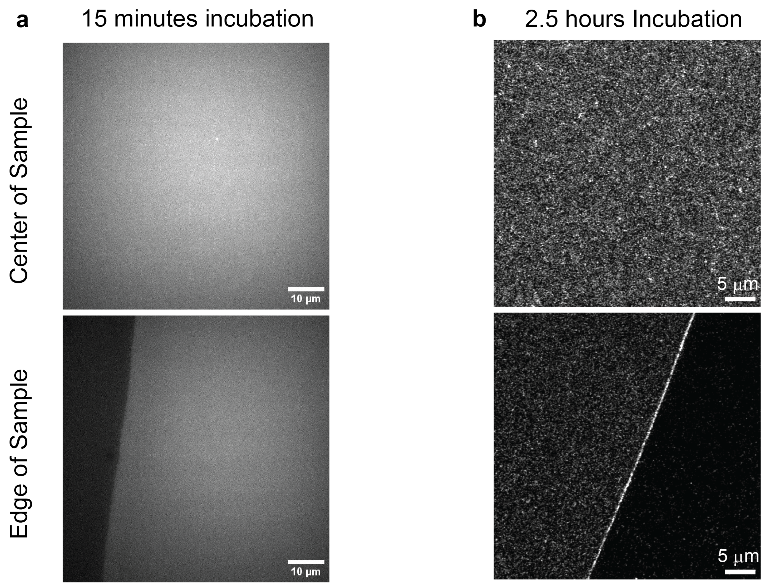

Figure S10: The 4xMS2 construct does not undergo aggregation or phase separation independent of the LAF-1 condensed phase. Under identical assay conditions to the condensate timecourses, but in the absence of LAF-1 protein, 4xMS2 condensates do not form either (a) initially or (b) after 2.5 hours of incubation. Micrographs in (a) were taken on a spinning disk confocal while the micrographs in (b) are from a Zeiss LSM980 with an Airyscan 2 detector. Scale bars correspond to: (a) 10 μm, (b) 5 μm.

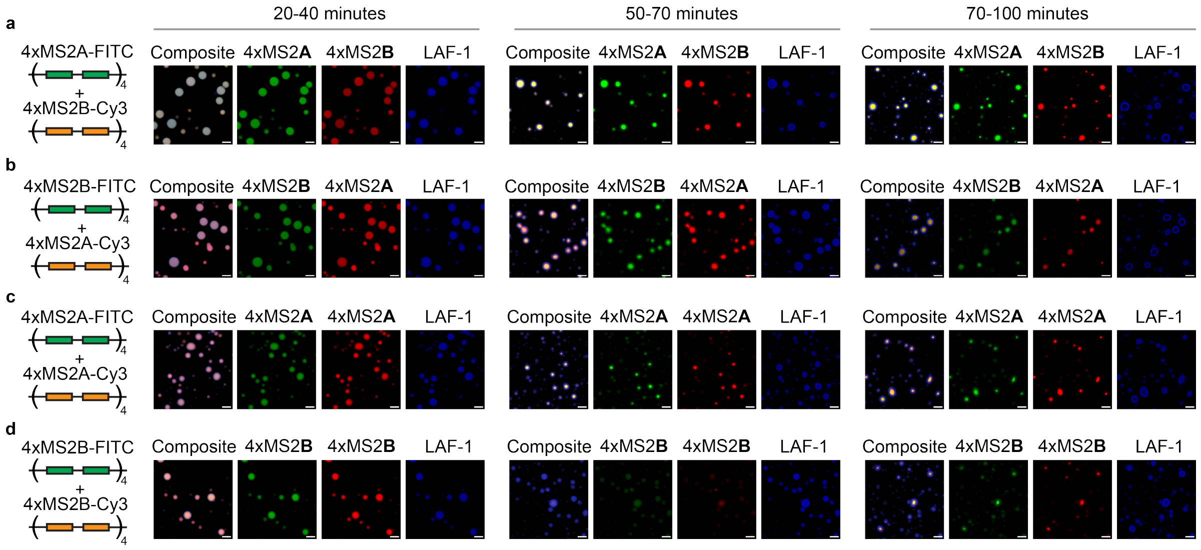

Figure S11: The second RNA phase transition does not depend on the specific base-pairing of the MS2 region. Separation between RNA-rich and protein rich phases is observed when both Part A and Part B of the MS2 hairpin sequence are present (a,b), but also when only tandem repeats of Part A are present (c), or when tandem repeats of Part B are present (d). Though the condensates begin as homogeneous protein-RNA droplets, separation between protein- and RNA-rich phases occurs over 50-90 minutes in all cases. This suggests the full MS2 hairpin on a single RNA strand is helping to prevent coalescence of RNA within the condensed phase. The Green channel indicates RNA labeled with fluorescein while the Red channel indicates RNA labeled with Cy3. Blue channel corresponds to LAF-1 protein. Micrograph intensity limits were set to be the same across a given channel for all time points and constructs. Scale bar is equal to 5 μm in all panels.

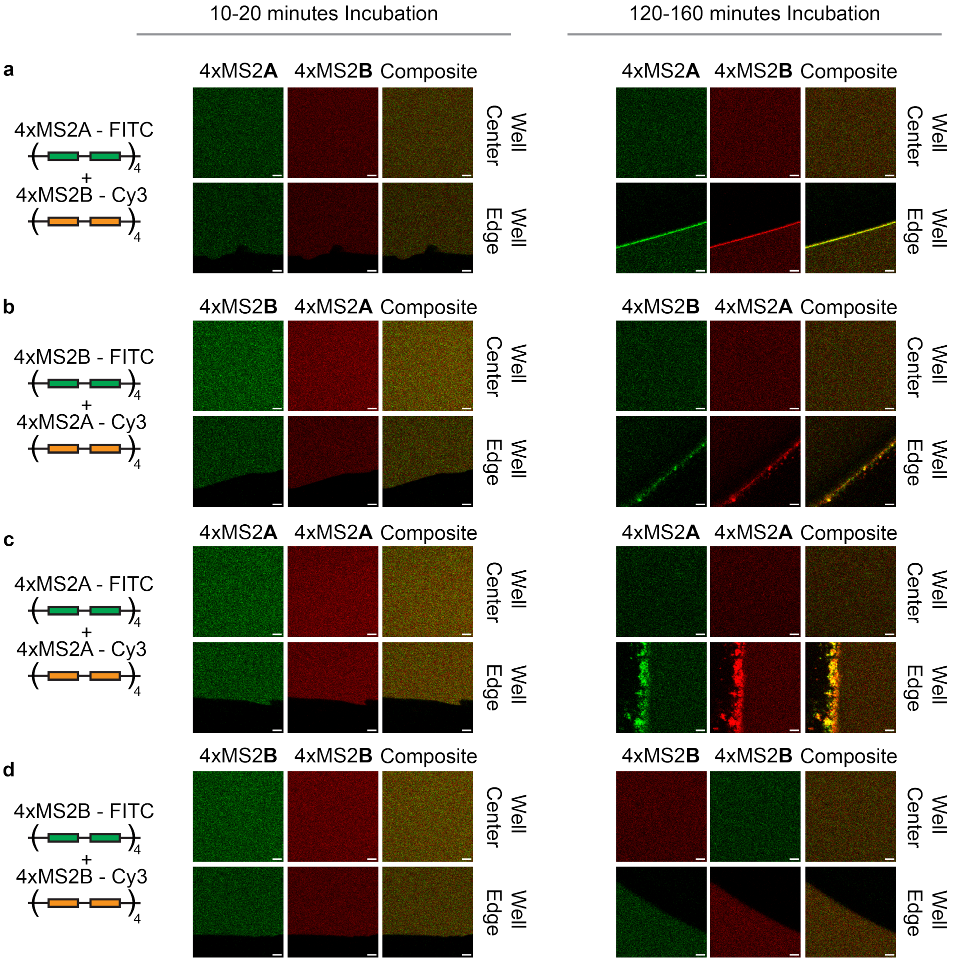

Figure S12: The different combinations of MS2 single-stranded sequences do not undergo phase separation or aggregation in the absence of LAF-1. Combining tandem repeats of (a) Part A and Part B of the MS2 hairpin sequence, (b) part A and part B with flipped fluorescent labels, (c) only Part A of the MS2 hairpin sequence, or (d) only part B of the MS2 hairpin sequence do not form aggregates in the absence of LAF-1 either initially or over 2 hours. Green or left hand channel indicates RNA labeled with fluorescein while the Red or center channel indicates RNA labeled with Cy3. Some aggregation can be seen at the well edge after 2 hours but is not seen in the bulk. Scale bar is equal to 5 μm in all panels.

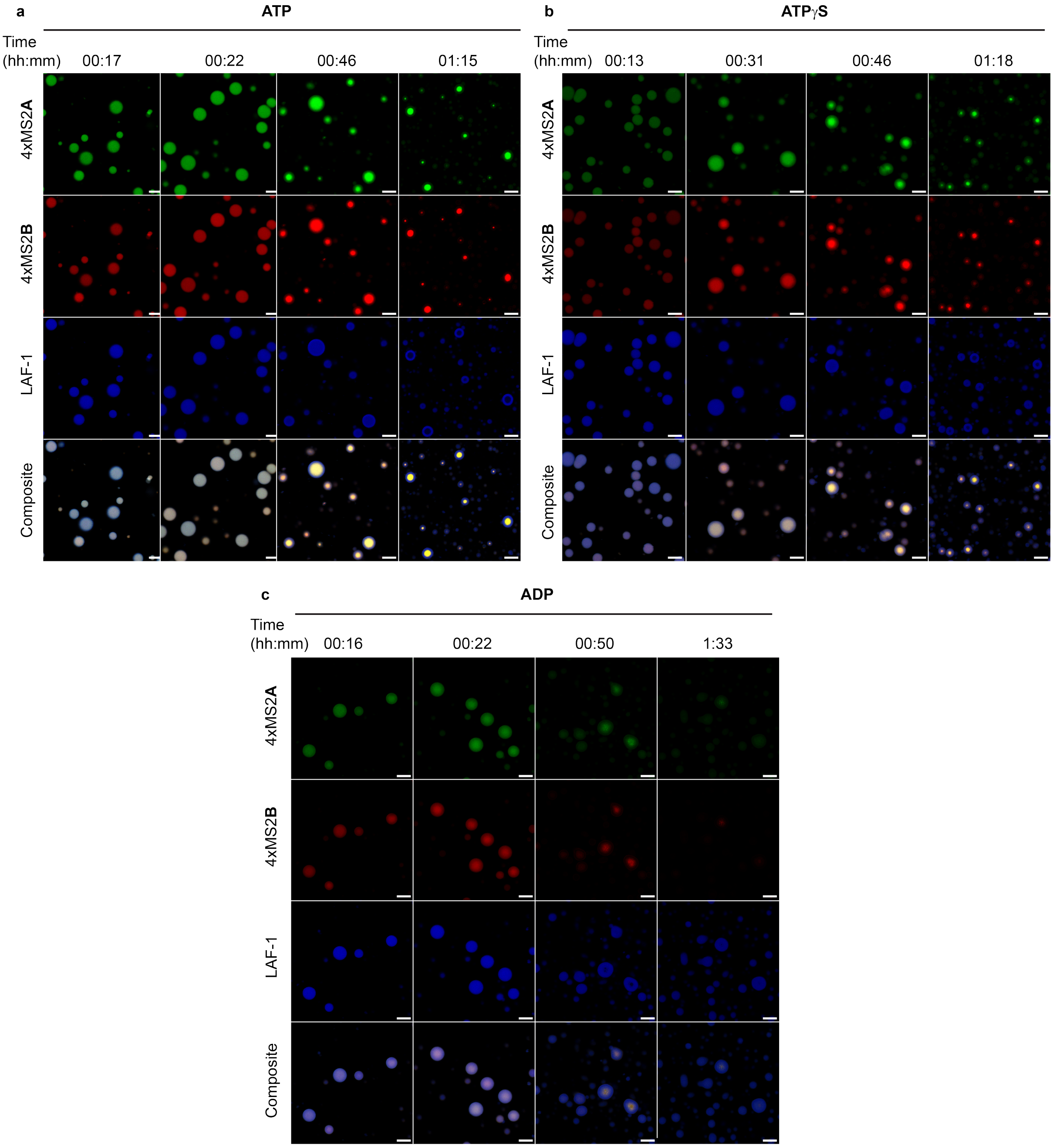

Figure S13: The secondary RNA phase transition occurs independent of LAF-1's ATPase activity. Snapshots of different fields of view of 4xMS2A, 4xMS2B, LAF-1 co-condensates imaged over 90 minutes. Systems were initialized in the presence of 1.6 mM (a) ATP, (b) ATPγS, or (c) ADP. A secondary RNA phase transition can be seen in the presence of each of the nucleotides. For ADP, RNA intensity within the condensed phase decreases over time, indicating some RNA departure from the condensed phase. Micrograph intensity limits were set to be the same across a given channel for all time points and nucleotide conditions. Scale bar is equal to 5 μm in all panels.
